# Short-video platforms as sources of atherosclerosis information: A cross-sectional content analysis

**DOI:** 10.1097/MD.0000000000045006

**Published:** 2025-10-03

**Authors:** Qiankuan Li, Lu Jin, Kaihang Shi, Xiaoran Zheng

**Affiliations:** a Department of Traditional Chinese Medicine, Handan Fukang Hospital, Handan, Hebei, China; b Zhongshan Institute for Drug Discovery, Shanghai Institute of Materia Medica, Chinese Academy of Sciences, Zhongshan, Guangdong, China; c Department of Dermatology and Venereology, Affiliated Hospital of Chengde Medical University, Chengde, Hebei, China; d Department of Internal Medicine, Handan Fukang Hospital, Handan, Hebei, China.

**Keywords:** atherosclerosis, digital health, health communication, health information quality, short video, social media

## Abstract

In recent years, short videos have shown significant promise in spreading health-related content. Yet, to the best of our knowledge, there has been no research that has evaluated the content and quality of atherosclerosis-related videos on short-video platforms. The goal of this research was to evaluate the content and quality of atherosclerosis-related videos on short-video platforms. We searched 4 platforms using predefined keywords (atherosclerosis, atherosclerotic disease, arteriosclerosis, arterial occlusion or vascular occlusion). We collected the top 50 videos per term on each platform. Data were collected on TikTok, Kwai, Rednote, and Bilibili from December 31, 2024 to January 8, 2025. Two independent researchers evaluated the content and quality of these videos by measures of Journal of the American Medical Association score, Global Quality Scale, modified DISCERN, and Patient Education Materials Assessment Tool (PEMAT). The data analysis was performed using SPSS and GraphPad Prism. Descriptive statistics were produced, and comparisons were made between different groups. The relationship between quantitative variables was examined using Spearman correlation analysis. A total of 764 suitable videos were selected for in-depth analysis, with the majority focusing on disease-related information (n = 670, 87.7%). The primary contributors were medical professionals (n = 546, 75.1%). The videos attained a mean Journal of the American Medical Association score of 1.8 (standard deviation [SD] 0.6), a Global Quality Scale rating of 3.1 (SD 0.8), and an modified DISCERN score of 2.7 (SD 0.6). They also had a PEMAT-Understandability score of 84.2% (SD 11.6%) and a PEMAT-Actionability percentage of 70% (SD 39.6%). The content shared by medical professionals and the videos that included information about illnesses were typically of superior quality and attracted significantly more engagement. Content related to treatment received more likes, comments, saves, and shares than other topics (*P* < .01). A significant positive relationship was found between the number of likes, comments, saves, and shares. Furthermore, the duration of the videos, the time elapsed since they were uploaded, and the follower count were all positively linked to both the popularity and perceived quality of the videos (*P* < .001). While short-video platforms host substantial content, the overall quality is suboptimal and requires systematic improvement and professional oversight.

## 1. Introduction

Atherosclerosis represents the primary pathological driver of ischemic heart disease, ischemic stroke, and peripheral arterial disease.^[1]^ Globally, ischemic heart disease and stroke are the first and third leading causes of mortality, respectively. In 2013, they accounted for a death rate of 247.9 per 100,000 individuals, representing 84.5% of all cardiovascular deaths and 28.2% of deaths from all causes.^[2]^ Pathologically, atherosclerosis is marked by lipid deposition, infiltration of inflammatory cells, and accumulation of fibrous tissue within arterial walls, leading to plaque formation that narrows and stiffens arteries (a central process in most cardiovascular diseases). The progression of atherosclerosis extends over decades, often beginning silently in childhood and manifesting with severe or disabling outcomes at first clinical presentation.^[3,4]^ Notably, approximately 60% of men and 45% of women experience acute myocardial infarction as their initial symptom.^[5]^ The widespread prevalence of hypertension, smoking, dyslipidemia, diabetes, obesity, physical inactivity, and unhealthy dietary patterns continues to amplify its global burden, with many deaths occurring before hospital care, emphasizing the need for effective dissemination.^[6,7]^ Thus, prevention and early management of atherosclerosis and its risk factors remain a pressing public health priority, emphasizing the need for effective dissemination of health knowledge and promotion of self-care strategies.

Meanwhile, China’s short video industry has undergone rapid expansion, giving rise to major platforms such as TikTok, Kwai, Bilibili, and Rednote. These platforms have attracted vast user bases by leveraging creative formats, interactive features, and dynamic communication.^[8]^ TikTok has become the most widely used short video platform in recent years. As of January 2024, TikTok has 1.62 billion users.^[9]^ The platform has over 600 million daily active users (DAU) and is widely used not only for entertainment but also for educational and health-related content.^[10]^ By 2024, Kwai reported more than 700 million DAU and 800 million monthly active users (MAU).^[11]^ Rednote recorded 120 million DAU and 320 million MAU by mid-2024.^[12]^ Similarly, Bilibili’s third quarter of 2024 financial report showed an average of 107 million DAU and 348 million MAU.^[13]^ These platforms have become prominent health communication channels, offering keyword-based access and visually engaging content that fosters emotional resonance and potential behavior change.^[14]^

In an era when short video platforms have become central to public health communication, the credibility of the information they deliver has never been more critical. For a condition as prevalent and life-threatening as atherosclerosis, misinformation can obscure risk awareness, delay prevention, and jeopardize timely treatment. Yet, despite the vast audiences these platforms command, no systematic evaluation of atherosclerosis-related content has been conducted. To bridge this gap, we established a multidimensional framework (integrating Journal of the American Medical Association benchmark criteria [JAMA], Global Quality Scale [GQS], modified DISCERN [mDISCERN], and Patient Education Materials Assessment Tool [PEMAT]) to evaluate not only informational quality but also how source, topic, and presentation shape user engagement. By uncovering these dynamics, our study seeks to advance evidence-based health communication, improve public literacy, and provide platforms with actionable insights for strengthening content moderation and professional verification.

## 2. Methods

### 2.1. Ethical considerations

This study analyzed publicly available, non-identifiable short-video content and involved no interaction with human participants. In accordance with institutional policy, formal ethics review was not required. All procedures complied with the Terms of Service of TikTok, Kwai, Bilibili, and Rednote and with applicable data-use regulations. No personal accounts or identifiable user information were collected or stored.

### 2.2. Data retrieval and collection methods

This investigation was conducted from December 31, 2024 to January 8, 2025 on TikTok, Kwai, Rednote, and Bilibili. Videos were ranked according to each platform’s default algorithm, which may incorporate relevance, popularity, and proprietary factors. TikTok provides public descriptions of its recommendation system, built on artificial intelligence/machine learning and deep learning. Fundamentally, this system operates through mathematical modeling (establishing statistical correlations between user interactions and content attributes) rather than comprehending content meaning. The model calculates a composite recommendation score based on weighted user behavior and video parameters, subsequently distributing the highest-ranked content to users.^[15]^ In order to reduce algorithmic bias, we created a new account and searched keywords including “动脉粥样硬化” (atherosclerosis) or “动脉粥样硬化症” (atherosclerotic disease) or “动脉硬化” (arteriosclerosis) or “动脉堵塞” (arterial occlusion) or “血管堵塞” (vascular occlusion). We took several precautions to minimize algorithmic bias, including clearing browsing history before each search, using a fixed IP address, and avoiding any personal information linkage. This protocol simulated non-personalized, standardized initial recommendation states to enhance impartiality and comparability of collected results. Prior studies suggest that the majority of viewers typically focus on the first few pages of search results.^[16,17]^ Consequently, accounting for platform algorithms and user viewing patterns, we acquired and documented top 50 videos per search term on each platform. The selection criteria were videos that address atherosclerosis and videos available in either Chinese or English. The disqualification criteria included redundant material, promotional content, and videos that discuss unrelated subjects (such as other medical conditions or off-topic information). The analysis assessed multiple video attributes, such as title, likes, comments, saves, shares, posting date, duration, source, uploader’s followers, presentation format, and content focus.

### 2.3. Video material and standard assessment

JAMA benchmark criteria were used to evaluate accuracy and trustworthiness of health information on digital platforms. These criteria focus on the author’s qualifications, transparency of authorship, comprehensive disclosure, and the freshness of the content.^[18,19]^ The GQS is a 5-point Likert scale designed to assess the overall quality of a video, taking into account overall video quality and usefulness for patients. It includes 5 distinct metrics, with ratings ranging from 1, which signifies poor quality, to 5, denoting exceptional smoothness and excellence.^[20,21]^ The mDISCERN is a 5-item instrument in academic literature for assessing the quality and dependability of online resources.^[22,23]^ JAMA, GQS, and the mDISCERN scores are commonly used in various studies.^[24–26]^ To further assess the comprehensibility and practical applicability of these videos, this study also utilized the PEMAT created by the Agency for Healthcare Research and Quality, evaluates health educational materials by assessing both PEMAT-Understandability (PEMAT-U) and PEMAT-Actionability (PEMAT-A).^[27]^ The specific scoring guidelines for the mentioned evaluation systems are available in Tables S1–S4, Supplemental Digital Content, https://links.lww.com/MD/Q216.

Two raters (XZ, QL) independently scored each video, disagreements were resolved by discussion with a third author (LJ), and inter/intra-rater reliability was assessed using intraclass correlation coefficient (ICC). This setup enabled us to compute the ICC, which was used to evaluate inter-rater and intra-rater reliability. Reliability is categorized as deficient (ICC < 0.50), average (ICC = 0.50–0.75), satisfactory (ICC = 0.75–0.90), or exceptional (ICC > 0.90) (25). Both researchers maintained good concordance in their evaluations of JAMA scores (ICC = 0.931, 95% confidence interval [CI] 0.921–0.940), GQS scores (ICC = 0.946, 95% CI 0.938–0.953), mDISCERN scores (ICC = 0.881, 95% CI 0.863–0.896), PEMAT-U (ICC = 0.886, 95% CI 0.869–0.901), and PEMAT-A (ICC = 0.924, 95% CI 0.912–0.934). The consistency within a single rater was notably high across all metrics (JAMA score = 0.976, GQS score = 0.884, mDISCERN score = 0.869, comprehensibility = 0.898, and applicability = 0.846). The internal agreement for each evaluator was also remarkably strong (JAMA score = 0.976, GQS score = 0.884, mDISCERN score = 0.869, comprehensibility = 0.898, and applicability = 0.846).

### 2.4. Statistical analysis

Data were analyzed using SPSS Statistics v22.0 (IBM Corp., Armonk) and GraphPad Prism v10.1.2 (Dotmatics, Boston). All raw values used to generate figures are available in Tables S1–S5, Supplemental Digital Content, https://links.lww.com/MD/Q216. Categorical variables were presented as frequencies and percentages. For numerical data that followed a normal distribution, means and standard deviation were utilized. Comparisons between 2 groups were conducted using either the Student *t*-test or the Mann–Whitney *U*-test. For comparisons involving 3 or more groups, 1-way analysis of variance was employed. The relationships between quantitative variables were assessed through Spearman correlation analysis. A *P* value <.05 was considered statistically significant.

## 3. Results

### 3.1. General characteristics of the videos

**Figure 1.**
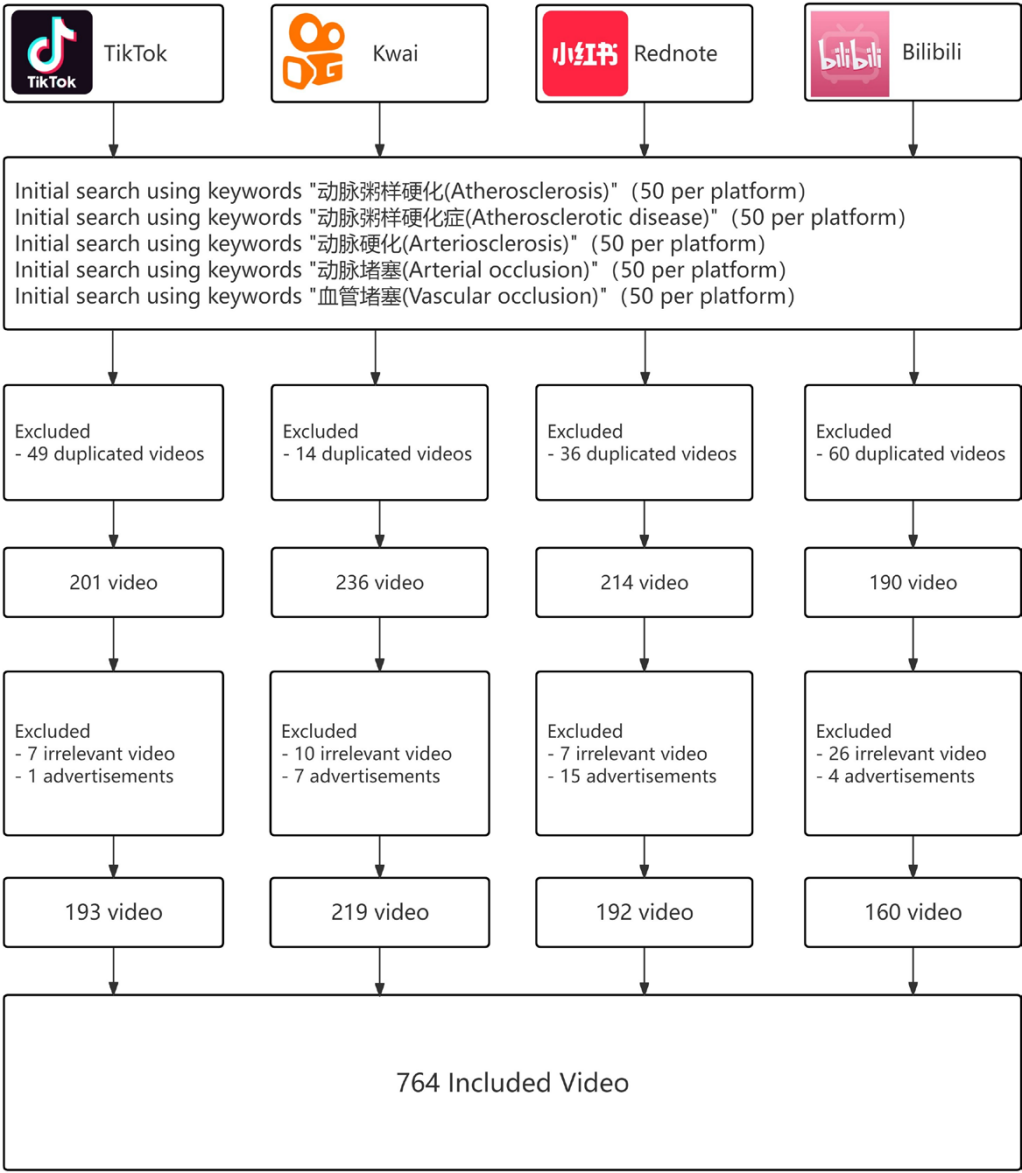
Flowchart of the study.

**Figure 2.**
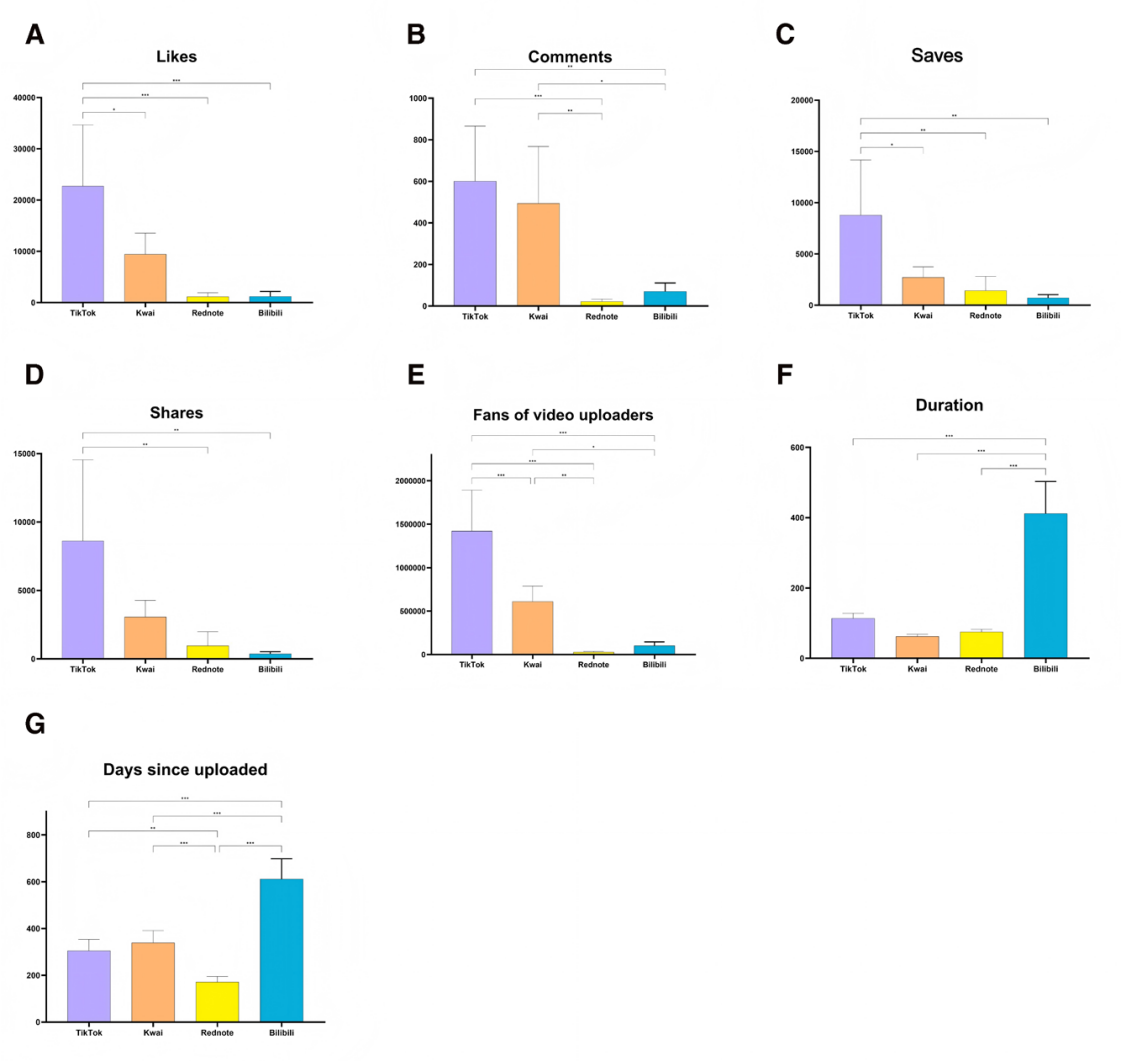
The general characteristics of the atherosclerosis-related videos: the bar chart shows (A) likes, (B) comments, (C) saves, (D) shares, (E) followers of video uploaders, (F) duration, and (G) days since upload. *P < .05; **P < .01; ***P < .001.

**Figure 3.**
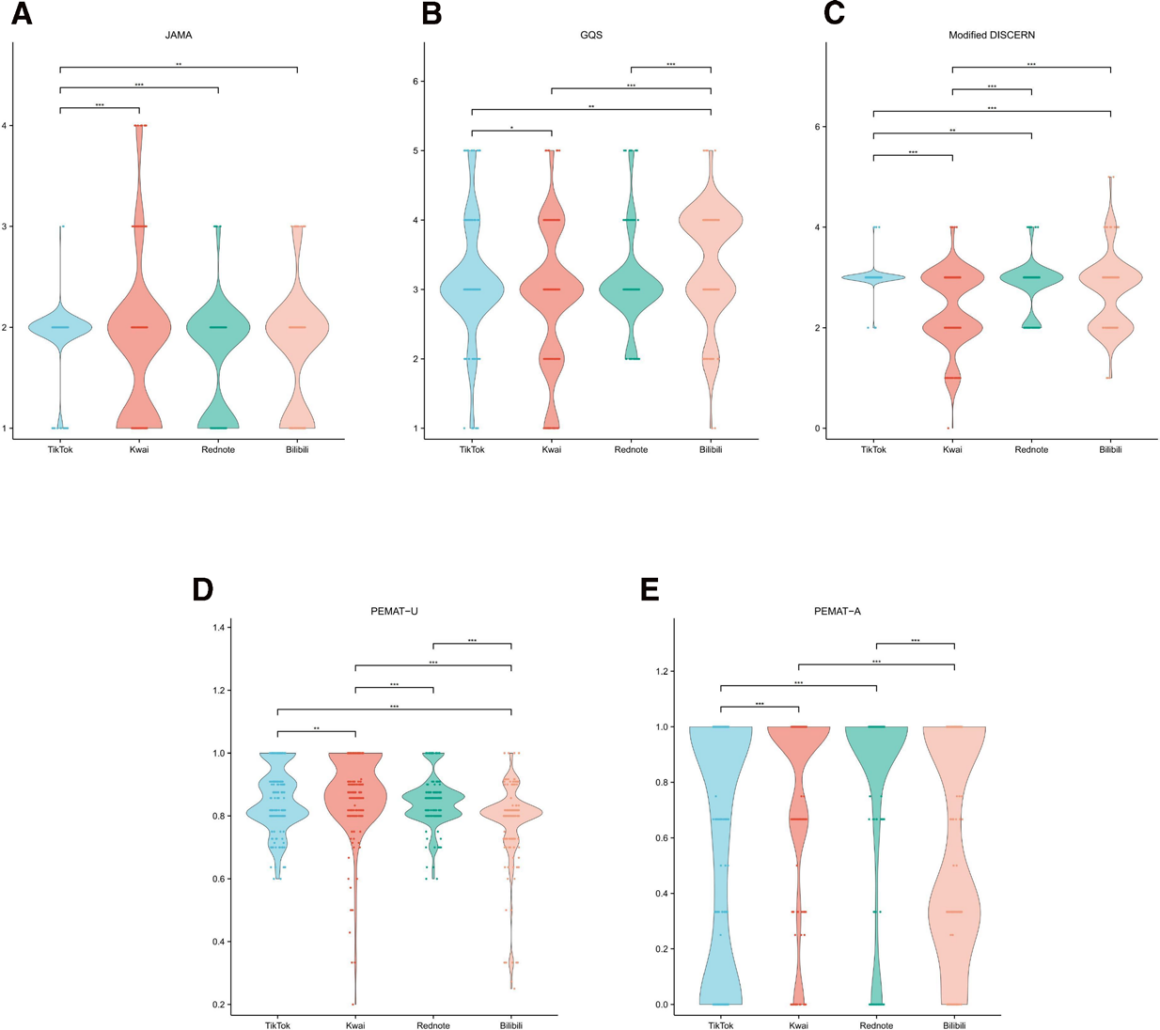
The scores of the atherosclerosis-related videos: the violin plots show (A) JAMA score, (B) GQS score, (C) mDISCERN score, (D) PEMAT-U score, and (E) PEMAT-A score. *P < .05; **P < .01; ***P < .001; ns, not significant. GQS = Global Quality Scale, JAMA = Journal of the American Medical Association, mDISCERN = modified DISCERN, PEMAT-A = Patient Education Materials Assessment Tool-Actionability, PEMAT-U = Patient Education Materials Assessment Tool-Understandability.

After removing duplicates, advertisements, and irrelevant content from a total of 1000 videos, 764 atherosclerosis-related ones were included for further analysis, with the process outlined in Figure 1. Table 1 and Figures 2 and 3 show that videos on TikTok received significantly higher user engagement metrics and follower counts than those on other platforms, with Kwai ranking second. Meanwhile, Bilibili hosted the longest-standing and lengthiest videos on atherosclerosis (*P* < .001). TikTok videos received higher GQS scores than those on Rednote, while Kwai scored lower (*P* < .001). The clarity and practicality of the content of the videos varied significantly among the platforms (*P* < .001).

**Table 1:** The general characteristics and scores of the atherosclerosis-related videos.

Parameters	Total (N = 764)	TikTok (n = 193)	Kwai (n = 219)	Rednote (n = 192)	Bilibili (n = 160)	*P* value
Duration (s), mean (SD)	151.65 (306.20)	113.67 (101.57)	62.19 (49.36)	74.96 (51.16)	411.96 (583.09)	<.001
Likes, mean (SD)	8993.23 (46,301.95)	22,731.44 (84,140.67)	9449.99 (30,807.44)	1156.82 (5310.54)	1200.04 (6249.32)	<.001
Comments, mean (SD)	313.62 (1472.13)	600.65 (1868.06)	493.62 (2057.53)	22.23 (79.72)	70.67 (259.23)	<.001
Saves, mean (SD)	3507.88 (20,326.32)	8788.34 (37,922.15)	2721.85 (7715.11)	1425.14 (9681.82)	713.52 (2019.43)	<.001
Shares, mean (SD)	3376.86 (22,057.00)	8624.06 (41,766.65)	3072.31 (9076.50)	953.94 (7326.38)	371.79 (988.94)	<.001
Days since upload, mean (SD)	345.17 (409.39)	304.85 (342.82)	338.94 (391.03)	171.02 (168.12)	611.29 (555.72)	<.001
Fans of video uploaders, mean (SD)	562,464.29 (1,897,908.36)	1,421,284.33 (3,316,232.45)	610,544.28 (1,341,423.59)	28,170.30 (55,566.48)	101,855.93 (279,175.36)	<.001
JAMA* score, mean (SD)	1.79 (0.59)	1.93 (0.29)	1.81 (0.78)	1.66 (0.55)	1.77 (0.61)	<.001
GQS† score, mean (SD)	3.09 (0.85)	3.13 (0.85)	2.84 (0.97)	3.08 (0.68)	3.37 (0.78)	<.001
mDISCERN‡ score, mean (SD)	2.71 (0.62)	2.99 (0.19)	2.37 (0.72)	2.83 (0.50)	2.71 (0.71)	<.001
PEMAT-U§, mean (SD)	84.20% (11.58%)	85.18% (9.97%)	87.57% (12.44%)	84.72% (7.85%)	77.77% (13.38%)	<.001
PEMAT-A‖, mean (SD)	70.09% (39.59%)	58.29% (44.50%)	78.73% (33.44%)	82.03% (35.23%)	58.18% (38.98%)	<.001

* Journal of American Medical Association.

† Global Quality Scale.

‡ Modified DISCERN.

§ Patient Education Materials Assessment Tool-Understandability.

‖ Patient Education Materials Assessment Tool-Actionability.

### 3.2. Origins and material of the videos

**Figure 4.**
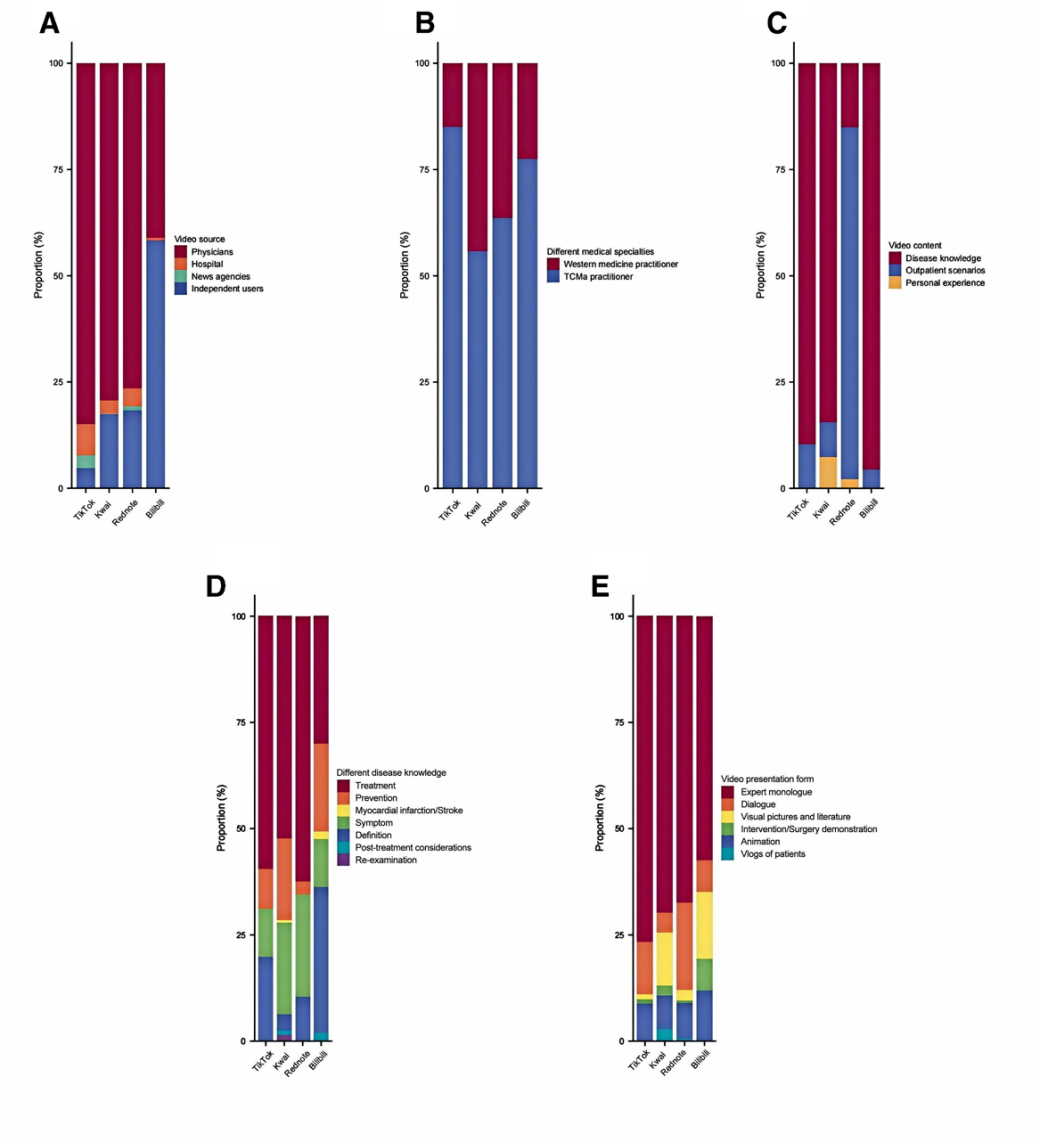
The stacked bar charts illustrate the distribution of multiple dimensions: (A) different sources, (B) Western medicine practitioners and TCM, (C) different contents, (D) different disease knowledge, and (E) video presentation forms. TCM = traditional Chinese medicine.

A comprehensive breakdown of the origins and material across the different platforms could be clearly captured in Figure 4 and Table S5, Supplemental Digital Content, https://links.lww.com/MD/Q216. The main contributors to the videos were doctors (546/764, 71.5%), and the content mainly consisted of disease knowledge (670/764, 87.7%) and outpatient scenarios (74/764, 9.7%). The disease knowledge in the videos primarily focused on treatment (392/764, 51.3%), prevention (97/764, 12.7%), and symptoms (131/764, 17.2%). Expert monologues (518/764, 67.8%), as well as dialogs (85/764, 11.1%) were the dominant presentation formats in the videos.

### 3.3. Factors affecting video appeal and effectiveness: source, content, and format

**Figure 5.**
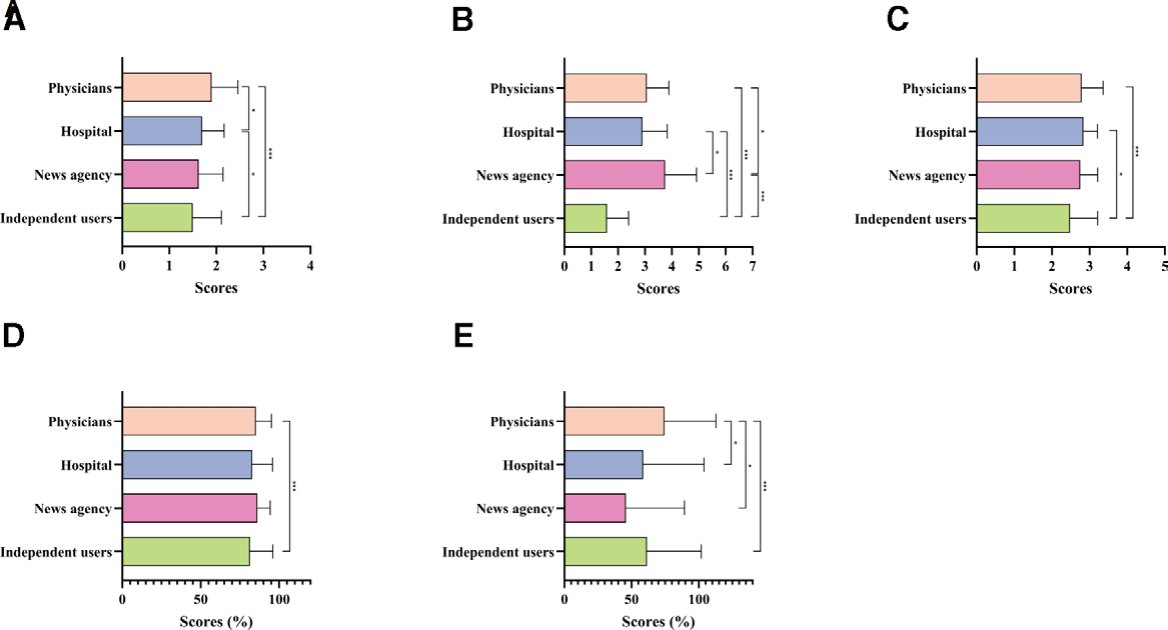
Comparative quality assessment of atherosclerosis short-videos with different sources. The bar chart shows quality evaluation of atherosclerosis short videos with different sources. (A) JAMA score; (B) GQS score; (C) mDISCERN score; (D) PEMAT-U; and (E) PEMAT-A. *P < .05; **P < .01; ***P < .001; ns, not significant. GQS = Global Quality Scale, JAMA = Journal of the American Medical Association, mDISCERN = modified DISCERN, PEMAT-A = Patient Education Materials Assessment Tool-Actionability, PEMAT-U = Patient Education Materials Assessment Tool-Understandability.

The criteria evaluations of JAMA for videos uploaded by doctors were superior to those of hospitals (*P* = .032; Fig. 5A), while videos from journalistic entities outperformed in GQS ratings compared to doctors, hospitals, and independent users (*P* = .021, *P* = .035, *P* < .001; Fig. 5B). The mDISCERN scores for doctors and hospitals were preferable to those of individual contributors (*P* < .001, *P* = .011; Fig. 5C). The PEMAT-U of doctor-uploaded videos demonstrated a marked increase over independent users (*P* < .001; Fig. 5D). The PEMAT-A of doctor-uploaded videos surpassed the scores of hospitals and news agencies by a slight amount (*P* = .026 and *P* = .036), and slightly higher than that of independent users (*P* < .001; Fig. 5E).

**Figure 6.**
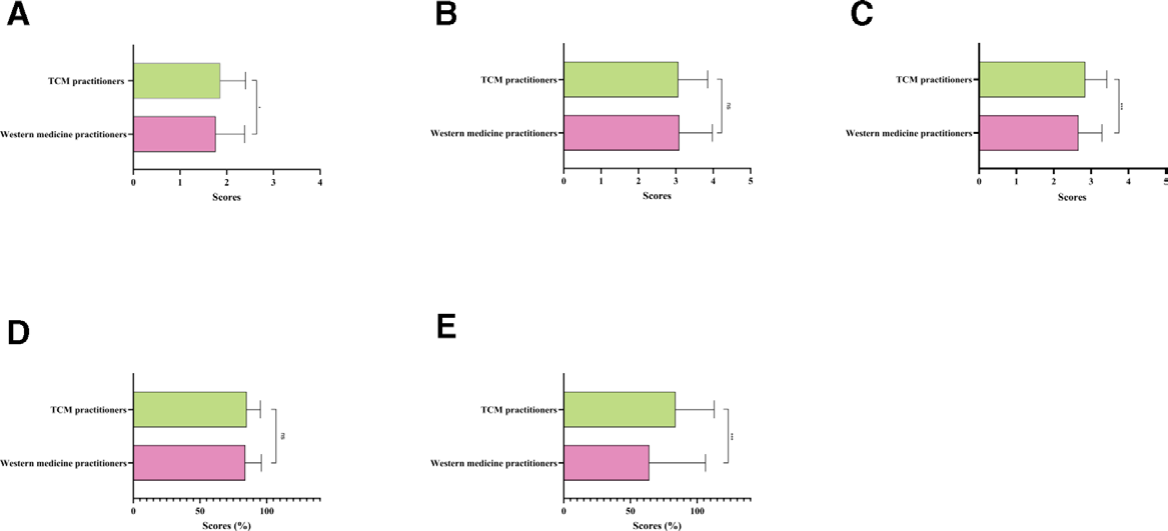
Comparative quality assessment of atherosclerosis short-videos with Western medicine and TCM. The bar chart shows quality evaluation of atherosclerosis short videos with Western medicine practitioners and TCM. (A) JAMA score; (B) GQS score; (C) mDISCERN score; (D) PEMAT-U; and (E) PEMAT-A. *P < .05; **P < .01; ***P < .001; ns, not significant. GQS = Global Quality Scale, JAMA = Journal of the American Medical Association, mDISCERN = modified DISCERN, PEMAT-A = Patient Education Materials Assessment Tool-Actionability, PEMAT-U = Patient Education Materials Assessment Tool-Understandability, TCM = traditional Chinese medicine.

Among videos uploaded by doctors, those from Western medicine practitioners had higher JAMA scores, mDISCERN scores, and PEMAT-A than those from traditional Chinese medicine practitioners (*P* = .018, *P* < .001, and *P* < .001; Fig. 6A, C and E). However, there were no notable differences in the GQS scores and the level of comprehensibility between the 2 groups (Fig. 6B and D).

**Figure 7.**
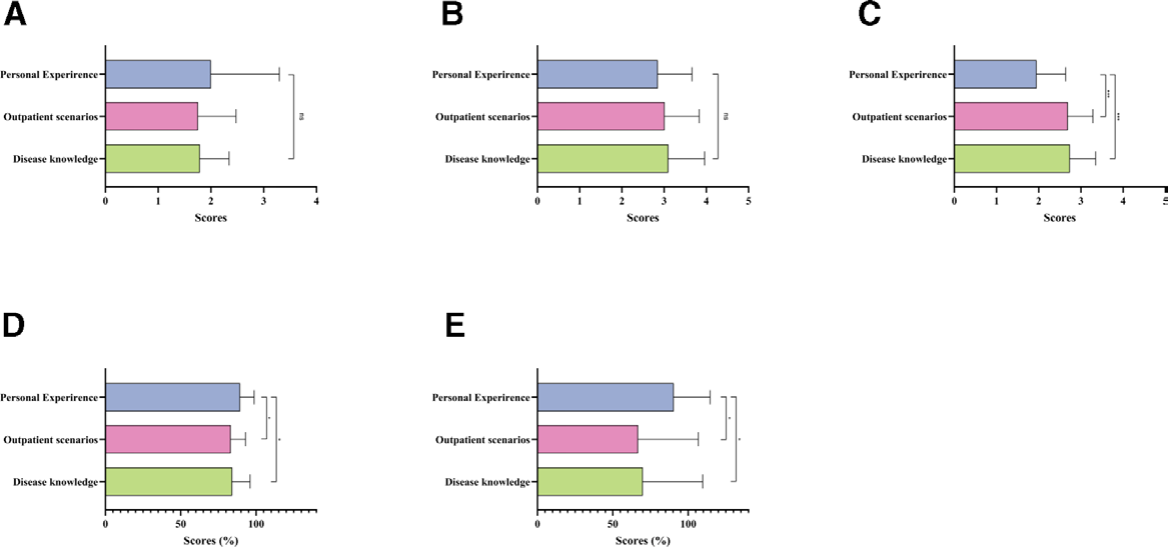
Comparative quality assessment of atherosclerosis short-videos with different contents. The bar chart shows quality evaluation of atherosclerosis short videos with different contents. (A) JAMA score; (B) GQS score; (C) mDISCERN score; (D) PEMAT-U; and (E) PEMAT-A. *P < .05; **P < .01; ***P < .001; ns, not significant. GQS = Global Quality Scale, JAMA = Journal of the American Medical Association, mDISCERN = modified DISCERN, PEMAT-A = Patient Education Materials Assessment Tool-Actionability, PEMAT-U = Patient Education Materials Assessment Tool-Understandability.

In the video content, the mDISCERN scores for disease knowledge and outpatient scenarios were substantially higher compared to personal experience-based videos (all *P* < .001; Fig. 7C). The PEMAT-U and PEMAT-A of personal experience videos were better than those of outpatient scenarios (*P* = .012 and *P* = .013; Fig. 7D and E) and disease knowledge (*P* = .048 and *P* = .022; Fig. 7D and E). There were no significant differences in JAMA and GQS across the 3 groups (Fig. 7A and B).

**Figure 8.**
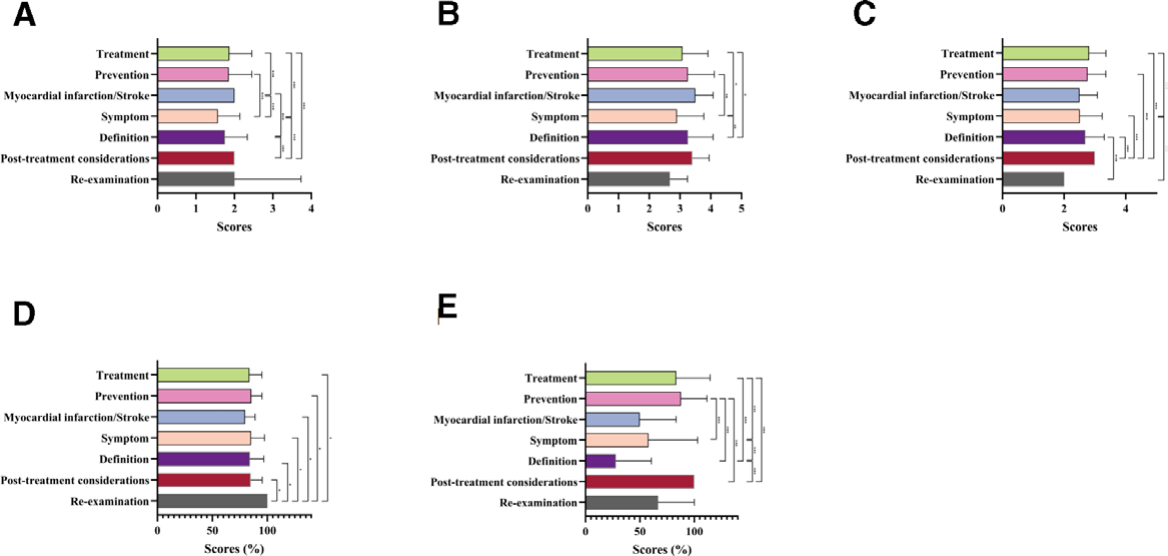
Comparative quality assessment of atherosclerosis short-videos with different disease knowledge. The bar chart shows quality evaluation of atherosclerosis short videos with different knowledge. (A) JAMA score; (B) GQS score; (C) mDISCERN score; (D) PEMAT-U; and (E) PEMAT-A. *P < .05; **P < .01; ***P < .001; ns, not significant. GQS = Global Quality Scale, JAMA = Journal of the American Medical Association, mDISCERN = modified DISCERN, PEMAT-A = Patient Education Materials Assessment Tool-Actionability, PEMAT-U = Patient Education Materials Assessment Tool-Understandability.

Treatment videos were most common and had higher JAMA scores than symptom videos (*P* < .001; Fig. 8A). Symptoms had lower GQS than treatment, prevention, and definition (*P* = .034, .002, .001; Fig. 8B). Posttreatment precautions had the highest mDISCERN, with treatment also outperforming symptoms (*P* < .001; Fig. 8C). Follow-up videos achieved the highest PEMAT-U (*P* < .001; Fig. 8D). For PEMAT-A, treatment and prevention exceeded symptoms and definition, while symptoms were higher than definition (*P* < .001; Fig. 8E).

**Figure 9.**
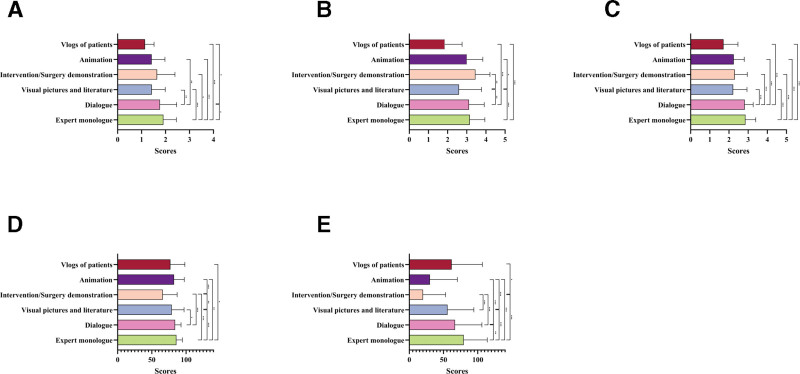
Comparative quality assessment of atherosclerosis short-videos with different presentation forms. The bar chart shows quality evaluation of atherosclerosis short-videos with different presentation forms. (A) JAMA score; (B) GQS score; (C) mDISCERN score; (D) PEMAT-U; and (E) PEMAT-A. *P < .05; **P < .01; ***P < .001; ns, not significant. GQS = Global Quality Scale, JAMA = Journal of the American Medical Association, mDISCERN = modified DISCERN, PEMAT-A = Patient Education Materials Assessment Tool-Actionability, PEMAT-U = Patient Education Materials Assessment Tool-Understandability.

Expert monologues consistently outperformed other formats. They achieved the highest JAMA scores (*P* < .05 to *P* < .001; Fig. 9A). For GQS, expert monologues and dialogs were higher than patient blogs (*P* < .001), whereas visual images and literature scored lower than other formats (*P* < .05; Fig. 9B). mDISCERN was also highest for monologues and dialogs (*P* < .001; Fig. 9C). In terms of PEMAT-U and PEMAT-A, expert monologues also performed better (*P* < .05 to *P* < .001; Fig. 9D and E).

Moreover, we examined the engagement levels of videos from various origins, themes, and presentation styles, as indicated by likes, comments, shares, and saves (Table 2). We found that videos featuring doctors attracted a higher number of likes, saves, and shares (*P* < .001). Content focused on disease information received more likes, comments, and saves (*P* < .001), while treatment-related videos were more frequently saved and shared (*P* < .001). Videos in the form of expert monologues were more likely to gain likes, comments, and saves (*P* = .004, *P* = .005, and *P* = .008).

**Table 2:** The popularity of videos from different sources with different contents and different presentation forms.

Variables	Likes	Comments	Saves	Shares
Video source				
Physician, mean (SD)	8617.65 (30,910.26)	322.87 (1464.37)	3352.18 (15,548.81)	2921.86 (11,998.439)
Hospital, mean (SD)	1590.2 (4601.09)	69.07 (178.09)	663.6 (2184.00)	467.5 (1199.38)
News agency, mean (SD)	27,798.13 (74,954.65)	1429.5 (3855.11)	6983 (19,032.50)	21,791.38 (60,872.85)
Independent user, mean (SD)	11,123.65 (79,534.79)	289.28 (1474.20)	4536.36 (32,650.76)	4665.98 (39,472.00)
Others, mean (SD)	448 (764.66)	63 (98.54)	277.9 (398.79)	301.1 (568.72)
*P* value	<.001	.004	<.001	<.001
Different medical specialties				
TCM* practitioner, mean (SD)	9421.44 (52,595.63)	324.23 (1632.07)	3102.53 (20,704.48)	3506.19 (25,466.62)
Western medicine practitioner, mean (SD)	8011.30 (26,851.04)	289.28 (1014.07)	4437.41 (19,443.10)	3080.29 (10,771.01)
*P* value	.0016	.139	.002	.004
Video content				
Disease knowledge, mean (SD)	9774.06 (48,632.72)	306.09 (1211.37)	3919.92 (21,664.88)	3780.31 (23,523.30)
Outpatient scenarios, mean (SD)	4048.14 (26,174.43)	445.12 (3027.59)	541.3 (2007.60)	439.03 (1273.51)
Personal experience, mean (SD)	1132.25 (1195.28)	79 (121.49)	681.15 (888.40)	731.2 (853.14)
*P* value	<.001	<.001	<.001	.002
Different disease knowledge				
Treatment, mean (SD)	7699.5 (26,937.83)	300.92 (1513.29)	3097.72 (10,957.39)	2753.59 (11,273.09)
Prevention, mean (SD)	23,690.78 (108,478.50)	700.82 (2323.31)	7984.25 (42,863.33)	8776.72 (52,048.85)
Myocardial infarction/stroke, mean (SD)	1322.50 (627.26)	80.5 (78.55)	1576.25 (1611.96)	1030.75 (891.66)
Symptom, mean (SD)	8380.73 (35,279.16)	280,24 (1222.22)	4567.89 (26,060.67)	4188 (20,835.75)
Definition, mean (SD)	3512.98 (16,798.21)	127,72 (458.67)	665.64 (1504.49)	716.51 (2731.50)
Posttreatment caveats, mean (SD)	2017 (2695.62)	23.4 (30.10)	451.4 (806.25)	760 (1201.90)
Reexamination, mean (SD)	985 (1461.08)	82 (98.05)	85 (77.89)	101 (101.84)
Others, mean (SD)	1213.18 (1745.31)	73.73 (96.55)	317.45 (468.24)	511 (687.66)
*P* value	.004	.006	<.001	<.001
Video presentation form				
Expert monologue, mean (SD)	11,868.72 (54,641.65)	396.13 (1701.04)	4801.61 (24,446.54)	4358 (25,611.23)
Dialogue, mean (SD)	874.78 (3132.80)	54.87 (210.66)	318.99 (1017.75)	356.02 (1170.72)
Intervention/surgery demonstration, mean (SD)	745.17 (1620.28)	57.68 (148.23)	595.88 (1292.27)	662.2 (1687.85)
Visual pictures and literature, mean (SD)	5014 (16,715.05)	285.29 (665.81)	1449.4 (4622.56)	824.75 (2134.59)
Animation, mean (SD)	7087.17 (32,215.24)	263.32 (1368.16)	1392.38 (6553.00)	3286.52 (20,921.02)
Vlog of patients, mean (SD)	1345.57 (2382.03)	49.71 (108.00)	499.43 (849.30)	1010.71 (2207.54)
Others, mean (SD)	984.83 (1185.03)	352.83 (778.49)	326.5 (369.20)	467.5 (401.16)
*P* value	.004	.005	.008	.039

* Traditional Chinese medicine.

### 3.4. Correlation analysis

As shown in Table 3, Spearman correlation (ρ) analysis revealed relationships between different video variables. The results indicated strong positive correlations between likes, comments, saves, shares, and follower numbers (all *P* < .001), with correlation coefficients ranging from 0.9 to 1. In contrast, likes, comments, saves, and shares had a weak positive correlation with video length and upload time (all *P* < .001).

**Table 3:** The correlation analysis between the video variables.

Variables	Likes	Comments	Saves	Shares
Likes				
ρ	1	0.937	0.953	0.936
*P* value	–*	<.001	<.001	<.001
Comments				
ρ	0.937	1	0.891	0.884
*P* value	<.001	–*	<.001	<.001
Collections				
ρ	0.953	0.891	1	0.950
*P* value	<.001	<.001	–*	<.001
Shares				
ρ	0.936	0.884	0.950	1
*P* value	<.001	<.001	<.001	–*
Duration				
ρ	0.122	0.141	0.209	0.123
*P* value	<.001	<.001	<.001	<.001
Days since uploaded				
ρ	0.156	0.156	0.175	0.205
*P* value	<.001	<.001	<.001	<.001
Fans				
ρ	0.709	0.653	0.607	0.601
*P* value	<.001	<.001	<.001	<.001

* Not applicable.

The JAMA scores exhibited a direct relationship with likes, comments, saves, shares, video length, and followers. Similarly, GQS demonstrated a positive association with comments, saves, video length, and follower count. The mDISCERN score was found to have a positive correlation with the length and follower numbers, but a negative correlation with the upload days. PEMAT-U was positively linked to follower count (*P* = .001) and inversely related to length. PEMAT-A, on the other hand, showed a negative correlation with the days since the video was uploaded (Table 4).

**Table 4:** The correlation analysis between video variables and the video quality.

Variables	JAMA*	GQS†	mDISCERN‡	PEMAT-U§	PEMAT-A‖
Likes					
ρ	0.162	0.111	0.077	0.056	0.027
*P* value	<.001	.002	.033	.119	.450
Comments					
ρ	0.143	0.125	0.060	0.054	0.015
*P* value	<.001	<.001	.095	.136	.674
Collections					
ρ	0.147	0.151	0.089	0.028	0.060
*P* value	<.001	<.001	.014	.439	.097
Shares					
ρ	0.144	0.112	0.058	0.068	0.087
*P* value	<.001	.002	.112	.062	.016
Duration					
ρ	0.156	0.419	0.250	−0.237	0.062
*P* value	<.001	<.001	<.001	<.001	.088
Days since uploaded					
ρ	−0.093	0.058	−0.120	−0.039	−0.177
*P* value	0.01	0.110	<0.001	0.278	<0.001
Fans					
ρ	0.242	0.124	0.148	0.117	0.068
*P* value	<.001	<.001	<.001	.001	.062

* Journal of American Medical Association.

† Global Quality Scale.

‡ Modified DISCERN.

§ Patient Education Materials Assessment Tool-Understandability.

‖ Patient Education Materials Assessment Tool-Actionability.

## 4. Discussion

### 4.1. Main findings

Health information is increasingly sought online, with an estimated 70% of internet users relying on the web as their primary source.^[28]^ Prior evaluations across conditions such as gallstones, chronic obstructive pulmonary disease, and diabetes have consistently revealed wide variation in the quality of online health videos.^[21,29,30]^ Extending this evidence, our study assessed the reliability and comprehensiveness of atherosclerosis-related content across 4 major platforms. TikTok emerged as the dominant platform, attracting the highest levels of engagement through saves, comments, and shares. Yet, overall content quality remained unsatisfactory, with substantial disparities between creators. Physicians were the primary contributors, and their content was consistently more accurate and trustworthy than that of lay users. Expert monologues represented the most common and effective format, providing clear and accessible disease knowledge. In terms of subject matter, treatment strategies, symptom recognition, and prevention were the most frequently addressed topics, with educational content outperforming anecdotal or clinic-based scenarios in both quality and audience reception. These findings underscore the central role of professional contributors and structured educational formats in shaping reliable online cardiovascular health communication.

### 4.2. The determinants of video appeal

Atherosclerosis videos generated substantial engagement, exceeding 1 million interactions, with some shared over 50,000 times, underscoring substantial public attention. Popularity, measured by likes, comments, saves, and shares, was highest for content from news agencies and physicians, reflecting both institutional credibility and public demand for clinically relevant information.^[28,29]^ More than half of physician videos focused on symptoms, consistent with rising interest in self-assessment and prevention. Atherosclerosis knowledge-focused videos attracted greater engagement than clinical scenarios or personal anecdotes, reflecting the limited scope and poor generalizability of the latter.

Engagement varied by subtopic: prevention dominated saves and shares, while acute events and follow-up care attracted less interest. This prioritization of proactive health aligns with evidence that endurance exercise reduces vulnerable plaques despite higher plaque burden,^[31]^ and that cardiac rehabilitation with >7000 daily steps yields greater plaque regression.^[32]^ Format also mattered: expert monologues proved most effective, while animations enhanced accessibility compared to simplistic styles. Likes strongly correlated with other metrics, and creator subscriber counts predicted higher engagement, underscoring the role of established audiences.

Therapeutic content drew particular attention. Combined dietary and pharmacological interventions delayed plaque progression and improved stability beyond drugs alone.^[33]^ Statins remain central, stabilizing plaques by lowering lipids and suppressing inflammation, as confirmed by angiographic studies of lovastatin.^[34]^ Non-statin agents (including ezetimibe, PCSK9 inhibitors,^[35]^ and omega-3 fatty acids) also show plaque-regressive effects.^[36]^ Antiplatelet therapy, notably aspirin, remains the first-line prophylaxis against recurrent stroke.^[37]^ Pharmacotherapy constitutes the cornerstone of management in advanced disease. For advanced disease, percutaneous coronary intervention restores coronary blood flow via stent implantation,^[38]^ while coronary artery bypass grafting bypasses complex lesions by creating alternative conduits, improving myocardial oxygenation and reducing cardiac event risk.^[39]^ Emerging anti-inflammatory and nanomedicine-based therapies reflect growing mechanistic insight.^[40,41]^

### 4.3. The factors correlated with video quality

Most atherosclerosis-related videos showed low-to-moderate ratings, suggesting widespread deficits in health information quality. Content from physicians, hospitals, and media outlets scored higher than that from individual contributors, reflecting professional training and reliance on current evidence. Expert monologues and dialogs were rated best, whereas vague or disorganized formats impaired clarity. Notably, some uploaders self-identified as “doctors” without verified credentials, raising concerns of impersonation to exploit perceived authority. This aligns with prior evidence that misinformation often originates from unverified sources.^[42]^ This underscores the critical need for public vigilance when accessing online health information.

The number of followers correlated positively with both video quality and engagement, indicating that perceived expertise and audience trust reinforce dissemination. By contrast, older videos received lower ratings, likely reflecting outdated information and reduced algorithmic visibility. Video length further influenced quality: longer videos were associated with higher ratings but reduced comprehensibility, possibly due to redundancy or attention fatigue.

Across platforms, TikTok demonstrated comparatively higher content quality, with physicians producing nearly 85% of health-related videos. Reports indicate the platform hosted over 35,000 certified healthcare professionals generating approximately 21,000 new posts daily; in the first half of 2023, health videos exceeded 100 million shares and 500 billion views.^[43]^ Nevertheless, quality remained poor overall: of 193 videos analyzed, only 2 reached a JAMA score >2 and 3 a mDISCERN score >3 in prior research. Similar deficiencies have been documented in chronic obstructive pulmonary disease,^[26]^ diabetes,^[44]^ cholelithiasis,^[18]^ and inflammatory bowel disease videos across multiple platforms indicating persistent deficits in accuracy, depth, and reliability of disease-specific health content.^[19]^

### 4.4. The recommendations based on our results

Short-video platforms have become central to health communication, particularly for high-burden diseases such as atherosclerosis. To improve quality and impact, creators should consider to prioritize clear, evidence-based content delivered through concise formats (expert monologues or dialogs) while avoiding excessive length. Independent users may adhere to scientific rigor, referencing up-to-date research and citing sources to enhance credibility and limit misinformation. Regular updates are also essential, given the rapid evolution of both medical knowledge and platform algorithms.

It is imperative for platforms to optimize their recommendation algorithms, by supporting content accuracy, providing timely medical updates, and monitoring user needs. Verification of contributors’ credentials is critical to curbing impersonation, while robust reporting systems and keyword optimization can improve credibility and information accessibility.

Public health strategies could help encourage users to exercise caution when engaging with online health content, prioritizing videos from verified professionals or institutions and considering credibility signals such as citation of authoritative sources and patterns of engagement. Importantly, short-video platforms should not be the sole source of health information; integration with professional websites, textbooks, and clinical consultation remains essential for comprehensive understanding.

### 4.5. Limitations

This study analyzed only Chinese-language videos from major domestic platforms. Thus, generalizability to other languages/platforms may be limited; the keyword list may have missed relevant content. International platforms such as YouTube and Instagram were excluded due to limited reach in the target population, though future work could incorporate cross-platform comparisons to enhance generalizability. The data collection window was brief (December 31, 2024–January 8, 2025), and searches were restricted to 5 keywords (“atherosclerosis,” “atherosclerotic disease,” “arteriosclerosis,” “arterial occlusion,” and “vascular blockage”). While these terms capture the core concepts, additional relevant keywords may have been overlooked.

A total of 764 videos were analyzed. Although this sample provides statistical validity, only the top 50 search results per keyword were included, potentially omitting less-exposed but high-quality content. Nevertheless, as most users focus on early search results, the dataset likely reflects the material that audiences most frequently encounter.

### 4.6. Conclusion

Short-video platforms have become prominent channels for atherosclerosis education, with physicians as the main contributors and expert lectures the dominant format. However, overall quality remains uneven. Enhancing professional verification, standardizing content, and strengthening platform-level moderation are essential to improve reliability and impact. For users, prioritizing verified medical sources and complementing online materials with evidence-based resources and clinical consultation will be key to obtaining reliable and comprehensive knowledge.

## Author contributions

**Conceptualization:** Xiaoran Zheng, Qiankuan Li, Lu Jin, Kaihang Shi.

**Data curation:** Xiaoran Zheng, Qiankuan Li, Lu Jin, Kaihang Shi.

**Formal analysis:** Xiaoran Zheng, Qiankuan Li, Lu Jin, Kaihang Shi.

**Funding acquisition:** Xiaoran Zheng.

**Investigation:** Xiaoran Zheng, Qiankuan Li, Lu Jin, Kaihang Shi.

**Methodology:** Xiaoran Zheng, Qiankuan Li, Lu Jin, Kaihang Shi.

**Project administration:** Xiaoran Zheng, Qiankuan Li.

**Resources:** Xiaoran Zheng, Qiankuan Li, Kaihang Shi.

**Software:** Xiaoran Zheng, Qiankuan Li, Lu Jin, Kaihang Shi.

**Supervision:** Xiaoran Zheng.

**Validation:** Xiaoran Zheng, Qiankuan Li, Lu Jin, Kaihang Shi.

**Visualization:** Xiaoran Zheng, Qiankuan Li, Lu Jin, Kaihang Shi.

**Writing – original draft:** Xiaoran Zheng, Qiankuan Li, Kaihang Shi.

**Writing – review & editing:** Xiaoran Zheng, Qiankuan Li, Lu Jin, Kaihang Shi.

## Supplementary Material
