# Supplementary material for: Short-video platforms as sources of atherosclerosis information: A cross-sectional content analysis
Source: Medicine (Baltimore). 2025 Oct 3;104(40):e45006. doi: 10.1097/MD.0000000000045006 (PMC12499849; doi:10.1097/MD.0000000000045006)
Supplement: Supplementary file 1 [file medi-104-e45006-s001.docx]

**Table S1. The *Journal of American Medical Association* (JAMA) benchmark criteria.**

| Criteria (1 point for each) | Description |
| --- | --- |
| Authorship | Author and contributor credentials and their affiliations should be provided |
| Attribution | Clearly lists all copyright information and states references and sources for content |
| Currency | Initial date of posted content and subsequent updates to content should be provided |
| Disclosure | Conflicts of interest, funding, sponsorship, advertising, support, and video ownership should be fully disclosed |

**Table S2. Description of the *Global Quality Score* (GQS) scale.**

| Scale | Description |
| --- | --- |
| Poor quality (1 point) | Poor quality and poor flow of the site, most information missing, not at all useful for patients |
| Generally poor quality (2 point) | Generally poor quality and poor flow, some information listed but many important topics missing, of very limited use to patients |
| Moderate quality (3 point) | Moderate quality, sub-optimal flow, some important information is adequately discussed but others poorly discussed, somewhat useful for patients |
| Good quality (4 point) | Good quality and generally good flow, most of the relevant information is listed, but some topics not covered, useful for patients |
| Excellent quality (5 point) | Excellent quality and excellent flow, very useful for patients |

**Table S3. Description of the modified DISCERN score.**

| Criteria  (1 point for each) | Description |
| --- | --- |
| 1 | Is the video clear, concise, and understandable? |
| 2 | Are reliable sources of information used? (i.e., publication cited, speaker is specialist) |
| 3 | Is the information presented balanced and unbiased? |
| 4 | Are additional sources of information listed for patient reference? |
| 5 | Are areas of uncertainty/controversy mentioned? |

**Table S4. The *Patient Education Materials Assessment Tool* (PEMAT)**

**Understandability.**

|  | Item | Response Options | Rating |
| --- | --- | --- | --- |
| Topic: Content | | | |
| 1 | The material makes its purpose completely evident. | Disagree=0, Agree=1 |  |
| Topic: Word Choice & Style | | | |
| 3 | The material uses common, everyday language. | Disagree=0, Agree=1 |  |
| 4 | Medical terms are used only to familiarize audience with the terms. When used, medical terms are defined. | Disagree=0, Agree=1 |  |
| 5 | The material uses the active voice. | Disagree=0, Agree=1 |  |
| Topic: Organization | | | |
| 8 | The material breaks or "chunks" information into short sections. | Disagree=0, Agree=1,  Very short material=N/A |  |
| 9 | The material’s sections have informative headers. | Disagree=0, Agree=1,  Very short material=N/A |  |
| 10 | The material presents information in a logical sequence. | Disagree=0, Agree=1 |  |
| 11 | The material provides a summary. | Disagree=0, Agree=1,  Very short material=N/A |  |
| Topic: Layout & Design | | | |
| 12 | The material uses visual cues (e.g., arrows, boxes, bullets, bold, larger font, highlighting) to draw attention to key points. | Disagree=0, Agree=1, Video=N/A |  |
| 13 | Text on the screen is easy to read. | Disagree=0, Agree=1,  No text or all text is narrated=N/A |  |
| 14 | The material allows the user to hear the words clearly (e.g., not too fast, not garbled). | Disagree=0, Agree=1,  No narration=N/A |  |
| Topic: Use of Visual Aids | | | |
| 18 | The material uses illustrations and photographs that are clear and uncluttered. | Disagree=0, Agree=1,  No visual aids=N/A |  |
| 19 | The material uses simple tables with short and clear row and column headings. | Disagree=0, Agree=1,  No tables=N/A |  |

Total Points: _____________

Total Possible Points: _____________

Understandability Score (%): _____________

(Total Points / Total Possible Points x 100)

**Actionability**

|  | Item | Response Options | Rating |
| --- | --- | --- | --- |
| 20 | The material clearly identifies at least one action the user can take. | Disagree=0, Agree=1 |  |
| 21 | The material addresses the user directly when describing actions. | Disagree=0, Agree=1 |  |
| 22 | The material breaks down any action into manageable, explicit steps. | Disagree=0, Agree=1 |  |
| 25 | The material explains how to use the charts, graphs, tables, or diagrams to take actions. | Disagree=0, Agree=1,  No charts, graphs, tables, diagrams=N/A |  |

Total Points: _____________

Total Possible Points: _____________

Actionability Score (%): _____________

(Total Points / Total Possible Points x 100)

**Table S5. The sources and content of the Atherosclerosis–related videos.**

| Variable | Total  (N=764), n (%) | TikTok  (n=193), n (%) | Kwai  (n=219), n (%) | Rednote  (n=192), n (%) | Bilibili  (n=160), n (%) | *P* value |
| --- | --- | --- | --- | --- | --- | --- |
| **Video source** |  |  |  |  |  | <.001 |
| Physicians | 546(71.47) | 164(84.97) | 173(79.00) | 147(76.56) | 62(38.75) |  |
| Hospital | 30(3.93) | 14(7.25) | 7(3.20) | 8(4.17) | 1(0.63) |  |
| News agencies | 8(1.05) | 6(3.11) | 0(0) | 2(1.04) | 0(0) |  |
| Independent users | 170(22.25) | 9(4.66) | 38(17.35) | 35(18.23) | 88(55.00) |  |
| Others | 10(1.31) | 0(0) | 1(0.46) | 0(0) | 9(5.63) |  |
| **Different medical specialties** |  |  |  |  |  | <.001 |
| Western medicine practitioner | 232(30.37) | 29(15.03) | 97(44.29) | 70(36.46) | 36(22.50) |  |
| TCMa practitioner | 532(69.63) | 164(84.97) | 122(55.71) | 122(63.54) | 124(77.50) |  |
| **Video content** |  |  |  |  |  | <.001 |
| Disease knowledge | 670(87.70) | 173(89.64) | 185(84.47) | 29(15.10) | 153(95.63) |  |
| Outpatient scenarios | 74(9.69) | 20(10.36) | 18(8.22) | 159(82.81) | 7(4.38) |  |
| Personal experience | 20(2.62) | 0(0) | 16(7.31) | 4(2.08) | 0(0) |  |
| **Different disease knowledge** |  |  |  |  |  | <.001 |
| Treatment | 392(51.31) | 115(59.59) | 109(49.77) | 120(62.50) | 48(30.00) |  |
| Prevention | 97(12.70) | 18(9.33) | 40(18.26) | 6(3.13) | 33(20.63) |  |
| Myocardial infarction/Stroke | 4(0.52) | 0(0) | 1(0.46) | 0(0) | 3(1.88) |  |
| Symptom | 131(17.15) | 22(11.40) | 45(20.55) | 46(23.96) | 18(11.25) |  |
| Definition | 121(15.84) | 38(19.69) | 8(3.65) | 20(10.42) | 55(34.38) |  |
| Post-treatment considerations | 5(0.65) | 0(0) | 2(0.91) | 0(0) | 3(1.88) |  |
| Re-examination | 3(0.39) | 0(0) | 3(1.37) | 0(0) | 0(0) |  |
| Others | 11(1.44) | 0(0) | 11(5.02) | 0(0) | 0(0) |  |
| **Video presentation form** |  |  |  |  |  | <.001 |
| Expert monologue | 518(67.80) | 148(76.68) | 150(68.49) | 128(66.67) | 92(57.50) |  |
| Dialogue | 85(11.13) | 24(12.44) | 10(4.57) | 39(20.31) | 12(7.50) |  |
| Visual pictures and literature | 59(7.72) | 2(1.04) | 27(12.33) | 5(2.60) | 25(15.63) |  |
| Intervention/Surgery demonstration | 20(2.62) | 2(1.04) | 5(2.28) | 1(0.52) | 12(7.50) |  |
| Animation | 69(9.03) | 17(8.81) | 17(7.76) | 16(8.33) | 19(11.88) |  |
| Vlogs of patients | 7(0.92) | 0(0) | 6(2.74) | 1(0.52) | 0(0) |  |
| Others | 6(0.79) | 0(0) | 4(1.83) | 2(1.04) | 0(0) |  |

^a^TCM: Traditional Chinese Medicine
